# Hypercalcaemia of Immobility in Critically Ill Patients: Case Series

**DOI:** 10.7759/cureus.43070

**Published:** 2023-08-07

**Authors:** Muhamad S Aljeaidi, Robert Palmer, Matthew H Anstey

**Affiliations:** 1 Medical School, The University of Western Australia, Perth, AUS; 2 Intensive Care Department, Sir Charles Gairdner Hospital, Perth, AUS; 3 School of Public Health, Curtin University, Perth, AUS

**Keywords:** hypercalcaemia, immobilisation, critical illness, intensive care unit, pamidronate

## Abstract

Significant hypercalcaemia can occur in intensive care unit (ICU) patients. Immobilisation hypercalcaemia has been infrequently reported after ICU admission. Patients, therefore, usually require extensive workup to rule out other common causes of hypercalcaemia, such as hyperparathyroidism. A case series of five patients who were diagnosed with hypercalcaemia due to immobilisation and received treatment with pamidronate between 2019 and 2023 were reported. The majority of cases were assessed as having hypercalcaemia due to immobilisation in the setting of low to normal parathyroid hormone levels, no suspicion of malignancy, and absence of other possible causative factors. Treatment with pamidronate started 10 to 60 days after hypercalcaemia was identified, and one or two doses of 30 mg of pamidronate were successful in resolving it. Immobilisation hypercalcaemia following ICU admission was uncommon but treatable with pamidronate.

## Introduction

In critically ill patients, metabolic bone disease is underappreciated even though critical illness has been linked to accelerated bone loss [1,2]. Metabolic bone disease can also be a manifestation of critical illness presenting as hypercalcaemia in its severe cases [1-3]. Increased bone resorption is evident among critically ill patients with markers up to eight times above the reference range from 24 hours of intensive care unit (ICU) admission [4,5].

Hypercalcaemia is not an uncommon finding in ICU patients [6]. In routine medical practice, hyperparathyroidism and malignancy-associated hypercalcaemia account for 90% of all cases [6]. There are a number of possible causes for this issue [6]. The workup of hypercalcaemia, hence, must exclude those conditions as well as vitamin D intoxication, medications such as thiazides and lithium, solid tumours, and endocrinopathies [6,7]. Immobilisation has been infrequently described as another cause of hypercalcaemia, especially in critically ill patients [8-10]. Although the underlying mechanism is not fully understood, it is thought that prolonged bed rest and increased osteoclast induction of bone resorption are contributing factors [10,11]. We sought to describe a cohort of patients with immobilisation hypercalcaemia in our ICU between 2019 and 2023 who received pamidronate as treatment. Medical records for the identified patients were recalled and screened for patient demographics, ICU admission details, hypercalcaemia onset and duration, laboratory tests, and any adverse effects of treatment.

## Case presentation

Case 1

A 75-year-old male with no significant past medical history presented to the emergency department with bilateral upper limb weakness following diarrhoeal illness two weeks prior. He was admitted under neurology and completed a lumbar puncture showing protein in the sample. Over two days, he progressively deteriorated, developing flaccid paralysis of all four limbs from the neck down and respiratory failure. He was then transferred to ICU for ventilation and airway protection; hence, a tracheostomy was inserted. His course had been complicated by two episodes of ventilator-associated pneumonia (requiring broad-spectrum antibiotics), new ECG changes, critical illness myopathy, and deranged liver function tests. His calcium levels gradually increased to 2.67 mmol/L (ionised calcium, 1.34 mmol/L) on the 23rd day of ICU admission. An appropriate workup was performed to investigate hypercalcaemia (Table 1). There was no suspicion of hyperparathyroidism or cancer-related hypercalcaemia. After a thorough workup, the patient was diagnosed with hypercalcaemia due to immobilisation. The patient received 30 mg of intravenous (IV) pamidronate over 24 hours on day 36 after hypercalcaemia was noted. The laboratory tests were normalised five days later. The patient was eventually transferred to inpatient rehabilitation services for further management.

Case 2

A 67-year-old male with high-grade bladder cancer was admitted for elective radical cystoprostatectomy, pelvic lymph node dissection, and formation of an ileal conduit. He had a significant past medical history with type 2 diabetes mellitus (T2DM), hypertension, hypercholesterolaemia, and coronary artery disease requiring previous stents. After the surgery, he was admitted to the high-dependency unit for a day but then stepped down to the ward. His stay was complicated by small bowel obstruction, and he underwent exploratory laparotomy and adhesiolysis. He developed severe aspiration pneumonia during induction for the surgery. The surgery, however, was completed; the patient remained intubated and was admitted to the ICU post-operation. He required ECMO for eight days for respiratory failure before a tracheostomy was performed. His ICU stay was later complicated with abdominal sepsis, critical care myopathy, and complex multidrug-resistant pathogens. After three months in ICU, his adjusted calcium levels gradually increased to 2.74 mmol/L in the setting of suppressed parathyroid hormone (PTH). He was on total parenteral nutrition (TPN), which was thought to be contributing to it. Hypercalcaemia was initially managed with volume expansion and adjustment of his TPN, but this failed to correct calcium levels. After a thorough workup, he was diagnosed with hypercalcaemia due to immobilisation and received one dose of 30 mg of IV pamidronate (Table 1). Calcium levels were corrected four days later, but he had transient asymptomatic hypocalcaemia, which self-resolved within 10 days. The patient was then admitted to the long-term inpatient stay unit to optimise his physical condition and ongoing rehabilitation.

Case 3

A 47-year-old male with a history of high alcohol intake, hypercholesterolaemia, and T2DM (on metformin and sitagliptin) was transferred from a regional hospital for ICU management with life-threatening pancreatitis in the background of previous acute alcoholic pancreatitis. He developed type 1 respiratory failure in the setting of abdominal compartment syndrome and a significantly raised intra-abdominal pressure. He was intubated for desaturation, and a tracheostomy was later performed. He was noted to be hypercalcaemic (adjusted calcium, 2.93 mmol/L; ionised calcium, 1.49 mmol/L), and after further investigations, he was diagnosed with hypercalcaemia due to immobilisation (Table 1). He received one dose of 30 mg of IV pamidronate. The patient was then transferred to the ward for further management. Calcium levels were still elevated 10 days later. Another dose of 30 mg of IV pamidronate was given in the ward. The calcium levels were normalised although he developed a transient asymptomatic hypocalcaemia, which self-resolved. The patient was discharged home two months later with outpatient and endocrine follow-up plans.

Case 4

A 63-year-old male with obesity and hypertension was transferred from a regional hospital for perforated sigmoid diverticulum and septic shock, requiring vasopressor support during the emergency transfer; hence, he was admitted to the ICU on arrival. He underwent a laparotomy, insertion of bilateral rectus sheath catheters, and formation of end colostomy. He was extubated on day 2 post-surgery and weaned off vasopressors. However, his ICU stay was complicated by intra-abdominal collection and rapid atrial fibrillation and atrial flutter. This was further complicated by pneumonia, retroperitoneal haemorrhage, and line sepsis. After 40 days in ICU, his adjusted calcium levels gradually increased to 3.26 mmol/L (ionised calcium, 1.6 mmol/L) in the setting of normal PTH levels (Table 1). He was initially managed with IV fluids, furosemide and calcitonin, which were not effective. After ruling out other causes, he was diagnosed with hypercalcaemia due to immobilisation and received 60 mg of IV pamidronate 10 days later. This successfully resolved the hypercalcaemia. When the patient stabilised, he was transferred to the ward for further management. He was then discharged a month later with rehabilitation.

Case 5

A 55-year-old male with T2DM and hypertension and a recent history of sleeve gastrectomy for morbid obesity developed a breakdown of the wound at the gastroesophageal junction and presented to the hospital with profound septic shock requiring ICU admission. He underwent initial emergency laparotomy, washout, and multiple drain insertion. Over the course of his admission, he continued to have issues with recurrent intraperitoneal and retroperitoneal collections due to persistent defects at the gastroesophageal junction. In addition, he had recurrent intra-abdominal sepsis requiring vasopressor support and broad-spectrum antibiotics. After two months, he was noted to be hypercalcaemic (adjusted calcium, 3.13 mmol/L; ionised calcium, 1.71 mmol/L), and after further investigations, he was diagnosed with hypercalcaemia due to immobilisation (Table 1). He received one dose of 30 mg of IV pamidronate, and his calcium levels were corrected a few days later. His stay was further complicated by nosocomial pneumonia, requiring increasing ventilatory support. In the context of his ongoing intra-abdominal sepsis and severe critical illness weakness, the decision was for palliation. The patient passed away in the presence of his loved one.

**Table 1:** Laboratory values and summary details of patients with hypercalcaemia TPN, total parenteral nutrition; PTH, parathyroid hormone; ALP, alkaline phosphatase

Parameter (reference range)	TPN (Y/N)	Cancer (Y/N)	Normal thyroid (Y/N)	Adj. Ca (pre), (2.10-2.60)	Adj. Ca (post), (2.10-2.60)	Adj. Ca (1 week), (2.10-2.60)	Adj. Ca (1 month), (2.10-2.60)	Ionised Ca (pre), (1.12-1.32)	eGFR (pre), (>60)	eGFR (post), (>60)	PTH (1.6-9)	Albumin (35-50)	ALP (30-110)	Vitamin-D 25 (OH), (>50)	Phosphate (0.75-1.50)	Magnesium (0.70-1.10)	Dose prescribed	Onset (days after ICU)	Days since onset Pamidronate started
Case 1	N	N	Y	2.65	2.54	2.54	2.58	1.34	>90	>90	1.8	41	134	50	1.08	0.83	30	23	36
Case 2	Y	N	Y	2.74	2.46	2.4	2.46	NA	48	56	0.5	27	182	60	1.32	0.73	30	90	30
Case 3	Y	N	Y	2.93	2.49	2.53	2.36	1.49	>90	>90	1.1	29	195	35	1.23	0.82	30 × 2	53	10
Case 4	N	N	Y	3.26	2.59	2.59	NA	1.6	40	>90	1.6	28	109	NA	1.08	0.87	60	40	15
Case 5	N	N	N	3.13	2.52	2.5	2.45	1.71	>90	>90	0.5	27	147	25	1.2	1.06	30	60	18

## Discussion

Immobilisation results in the loss of mechanical stress that is needed for bone formation and causes an influx of calcium due to bone resorption [6]. This results in a gradual increase in calcium levels, which causes a decrease in levels of PTH [6,12]. A previous case report of a renal patient noted a gradual increase in calcium levels starting approximately two months into admission [13]. Our patients had similar findings with the onset of hypercalcaemia, ranging from 23 to 90 days after ICU admission. In addition, all patients had low to normal PTH levels. All cases were assessed as having immobilisation hypercalcaemia in the setting of low to normal PTH, no suspicion of malignancy, and absence of other possible causative factors. Other studies have investigated N-telopeptide, vitamin D, phosphate, magnesium levels, and calciuria in the assessment of hypercalcaemia [6,12,14]. Those measurements can differentiate if the cause of hypercalcaemia is in fact immobilisation or other causes [12,15]. Vitamin D-dependent hypercalcaemia is associated with phosphate level changes [14]. All our patients had low to normal vitamin D and normal phosphate and magnesium levels, lowering the possibility of other causes of hypercalcaemia. We also note that the level of adjusted calcium ranged from (2.65-3.44 mmol/L), and total calcium ranged from (2.48-3.1 mmol/L), indicating mild to moderate hypercalcaemia [6]. Immobilisation causes a mild elevation in calcium that is often symptomless, unlike the high rise seen in malignancy-related hypercalcaemia [6]. As a result, initial treatment may start with IV fluids [16]. Medications that increase the excretion of calcium, such as furosemide, can be used simultaneously [7]. In addition, calcitonin and bisphosphonates inhibit bone resorption and can be used to treat immobilisation hypercalcaemia [7,16]. Calcitonin effect is transient and often used, while bisphosphonate’s effect starts [7,16]. Pamidronate (90 mg) and zoledronic acid (4 or 8 mg) are bisphosphonate agents that can be used to treat moderate to severe hypercalcaemia (corrected serum calcium, ≥3.00 mmol/L) [17]. In refractory hypercalcaemia, denosumab might be considered owing to its different mechanism of action and independence from patients’ renal function [5,13]. Majority of our patients did not have any symptoms of hypercalcaemia, and hence, the initial approach included watchful wait (cases 1, 3, and 5), volume expansion (cases 2 and 4), and furosemide (case 4). All patients then received pamidronate when initial management failed. Our patients received a lower dose of pamidronate when compared to other studies, and no major adverse effects and, specifically, no nephrotoxicity were reported. Massagli and Cardenas reported using doses of 60 and 90 mg of pamidronate in their patients [15]. In our three patients, hypercalcaemia was resolved after taking one dose of 30 mg of pamidronate for one patient and a total dose of 60 mg of pamidronate for the other two patients.

## Conclusions

In conclusion, hypercalcaemia is not an uncommon finding in ICU patients. Clinicians should consider all possible causes, including immobilisation hypercalcaemia, as ICU patients may be particularly prone to it. The present case series demonstrated that immobilisation hypercalcaemia can be successfully treated with lower IV pamidronate doses than previously described with no major adverse effects reported.
